# A systematic review and meta-analysis comparing the efficacy and safety of ciprofol (HSK3486) versus propofol for anesthetic induction and non-ICU sedation

**DOI:** 10.3389/fphar.2023.1225288

**Published:** 2023-09-25

**Authors:** Kuo-Chuan Hung, Jen-Yin Chen, Shao-Chun Wu, Po-Yu Huang, Jheng-Yan Wu, Ting-Hui Liu, Chien-Cheng Liu, I-Wen Chen, Cheuk-Kwan Sun

**Affiliations:** ^1^ School of Medicine, College of Medicine, National Sun Yat-sen University, Kaohsiung, Taiwan; ^2^ Department of Anesthesiology, Chi Mei Medical Center, Tainan, Taiwan; ^3^ Department of Anesthesiology, Kaohsiung Chang Gung Memorial Hospital, Chang Gung University College of Medicine, Kaohsiung, Taiwan; ^4^ Department of Internal Medicine, Chi Mei Medical Center, Tainan, Taiwan; ^5^ Department of Nutrition, Chi Mei Medical Center, Tainan, Taiwan; ^6^ Department of Psychiatry, Chi Mei Medical Center, Tainan, Taiwan; ^7^ Department of Anesthesiology, E-Da Hospital, I-Shou University, Kaohsiung, Taiwan; ^8^ Department of Anesthesiology, Chi Mei Medical Center, Liouying, Tainan City, Taiwan; ^9^ Department of Emergency Medicine, E-Da Dachang Hospital, I-Shou University, Kaohsiung, Taiwan; ^10^ School of Medicine for International Students, College of Medicine, I-Shou University, Kaohsiung, Taiwan

**Keywords:** ciprofol, meta-analysis, sedation, anesthetic induction, propofol

## Abstract

**Background:** Ciprofol (HSK3486) is a novel intravenous anesthetic agent that bears structural similarity to propofol and displays favorable pharmacodynamic characteristics such as rapid onset and offset. The meta-analysis aimed at comparing the efficacy and safety of ciprofol versus propofol in clinical practice.

**Methods:** Medline, EMBASE, Google Scholar, Cochrane Library were searched from inception to April 2023. The primary outcome was success rate of sedation/anesthetic induction and differences in sedation/induction time. The secondary outcomes included risks of hemodynamic instability, respiratory complications, and pain on injection, as well as recovery profiles, satisfaction score, and top-up dose requirement.

**Results:** Twelve RCTs (sedation: *n* = 6, anesthetic induction, *n* = 6, all conducted in China) involving 1,793 patients (age: 34–58 years) published from 2021 to 2023 were analyzed. Pooled results revealed no differences in success rate [risk ratio (RR) = 1, 95% confidence interval (CI): 0.99 to 1.01, I^2^ = 0%, 1,106 patients, *p* = 1] and time required for successful anesthetic induction/sedation [mean difference (MD) = 7.95 s, 95% CI: −1.09 to 16.99, I^2^ = 97%, 1,594 patients, *p* = 0.08]. The risks of top-up dose requirement (RR = 0.94, *p* = 0.48), cardiopulmonary complications [i.e., bradycardia (RR = 0.94, *p* = 0.67), tachycardia (RR = 0.83, *p* = 0.68), hypertension (RR = 1.28, *p* = 0.2), hypoxemia/pulmonary depression (RR = 0.78, *p* = 0.24)], and postoperative nausea/vomiting (RR = 0.85, *p* = 0.72), as well as discharge time (MD = 1.39 min, *p* = 0.14) and satisfaction score (standardized MD = 0.23, *p* = 0.16) did not differ significantly between the two groups. However, the ciprofol group had lower risks of hypotension (RR = 0.85, *p* = 0.02) and pain on injection (RR = 0.17, *p* < 0.00001) than the propofol group. The time to full alertness was statistically shorter in the propofol group (i.e., 0.66 min), but without clinical significance.

**Conclusion:** Our results demonstrated similar efficacy between ciprofol and propofol for sedation and anesthetic induction, while ciprofol was associated with lower risks of hypotension and pain on injection. Future studies are warranted to evaluate the efficacy and safety of ciprofol in pediatric or the elderly populations.

**Systematic Review Registration:** (https://www.crd.york.ac.uk/prospero/), identifier (CRD42023421278).

## 1 Introduction

Propofol, which is a potent intravenous hypnotic agent, is commonly used for anesthetic induction because of its rapid onset of action, a relatively low incidence of pharyngeal morbidity, as well as the ease of administering and monitoring (Joo and Perks, 2000; Chen et al., 2021). In addition, propofol is often the sedative agent of choice for painful diagnostic procedures (e.g., colonoscopy) where patients need to be cooperative with minimal movement. Propofol is particularly useful in outpatient settings due to its ability to achieve a fast recovery with a low risk of postoperative nausea/vomiting (PONV) (Abad-Santos et al., 2003; Sahinovic et al., 2018; Zhang et al., 2018; Dong et al., 2023). Propofol primarily exerts its pharmacological effects by activating the gamma-aminobutyric acid (GABAA)-receptor subunit β1, which in turn increases inhibitory synaptic transmission through the chloride channels, leading to anesthesia and sedation (Hansen, 2015). Despite the known clinical benefits, the use of propofol may be associated with cardiopulmonary depression in a dose-dependent manner, which can result in adverse events such as hypotension, bradycardia, and apnea (Coté et al., 2010; Sneyd et al., 2022). In the anesthesia setting, a previous study of 42,825 patients who underwent elective non-cardiac surgery found a significant correlation between post-induction hypotension and the risk of acute kidney injury (Maheshwari et al., 2018), highlighting the safety concern about propofol use. Besides, propofol administration is associated with injection pain (Euasobhon et al., 2016). These drawbacks have prompted the search for alternative anesthetic agents that can provide similar efficacy without compromising patient safety and comfort.

Ciprofol (HSK3486) is a novel intravenous anesthetic agent that bears structural similarity to propofol and displays favorable pharmacodynamic characteristics such as rapid onset and offset (Qin et al., 2017; Bian et al., 2021). Clinical studies have compared the efficacy and safety of ciprofol with those of propofol in patients receiving various elective surgery or sedative procedures (e.g., gastrointestinal endoscopy) (Teng et al., 2021; Chen et al., 2022a; Luo et al., 2022; Wu et al., 2022; Liang et al., 2023). Some studies reported no difference in success rate or time required for anesthetic induction/sedation between ciprofol and propofol, while ciprofol seems to have a more stable hemodynamic profile and a lower incidence of adverse events (Chen et al., 2022a; Wu et al., 2022). Despite the promising findings from previous clinical studies, the efficacy and safety of ciprofol in clinical practice have yet to be fully established due to the absence of large-scale randomized controlled trials (RCTs). The current meta-analysis aimed at assessing the efficacy and safety of ciprofol relative to propofol by combining the results of various studies. Moreover, the present investigation attempted to identify the probable origins of heterogeneity and inconsistencies across different studies to provide more accurate evaluations of the treatment outcomes.

## 2 Methods

The protocol for the present meta-analysis was officially registered on PROSPERO (CRD42023421278). The report of this meta-analysis adhered to the PRISMA (Preferred Reporting Items for Systematic Reviews and Meta-Analyses) criteria.

### 2.1 Data source and literature searches

Two investigators, working independently, systematically conducted a comprehensive literature search in databases including MEDLINE, EMBASE, Cochrane Library, and the Google Scholar. The search was performed from the inception of these databases up to 26 April 2023 with a specific focus being placed on articles that compared the efficacy and safety of ciprofol with those of propofol in patients receiving sedation or anesthetic induction. The search terms included: (“Sedation” or “Sedative” or “Deep sedation” or “procedural sedation” or “depression of consciousness” or “sedative” or “Conscious sedation” or “Moderate sedation” or “General anesthesia*” or “Anesthesia*” or “tracheal intubation” or “laryngeal mask airway” or “anesthetic induction”) and (“HSK3486”or “ciprofol”). To ensure an exhaustive search, a combination of controlled vocabulary and synonyms was employed, with no limitations on language or publication date. The search strategy for one of the databases, MEDLINE, is detailed in Supplemental Table S1. Furthermore, to identify potential additional references meeting the inclusion criteria, the investigators meticulously scrutinized the reference lists of all included studies and those of relevant reviews.

### 2.2 Eligibility criteria

The inclusion criteria used were as follows: (a) Population: individuals aged 18 years or above receiving surgeries or procedures under general anesthesia or sedation; (b) Intervention: the administration of ciprofol, either as a standalone agent or in combination with opioids, for the purpose of sedation or anesthetic induction; (c) Comparator: the use of propofol with or without opioid for sedation or anesthetic induction; (d) Outcomes: availability of details on the success rate of sedation/anesthetic induction, risk of hemodynamic instability, recovery profiles, or adverse events; and (e) Type of study: only RCTs were included.

The exclusion criteria were 1) studies that focused on patients in the intensive care unit (ICU) setting; 2) studies that had no control group (i.e., propofol not used); 3) pharmacokinetics studies; 4) number of patients in each group less than 20. The cutoff of <20 patients per group was chosen based on recommendations that RCT arms should have at least 20 subjects to ensure adequate statistical power (Julious, 2005); and 5) articles presented as reviews, case reports, conference abstracts, non-peer-reviewed articles, or letters. In cases where studies reported multiple subgroups with varying sample sizes, we only included arms with >20 patients and did not extract data from arms with <20 patients. Importantly, we did not exclude full studies from eligibility just because they had some smaller arms, as long as they also had arms with sufficient sample sizes that could be analyzed.

### 2.3 Studies selection and data extraction

The titles and/or abstracts of studies were screened separately by two reviewers to identify studies that potentially meet the predefined inclusion criteria, the full text of which were retrieved and independently assessed for eligibility by the two reviewers. Any disagreement on study eligibility between the two reviewers was settled by a discussion that may involve a third reviewer if necessary.

Relevant information, including the clinical setting (i.e., sedation or anesthetic induction), first author’ name, publication year, patient characteristics (e.g., gender), sample size, American Society of Anesthesiologists (ASA) physical status, episodes of hemodynamic instability (e.g., bradycardia), respiratory complications (i.e., hypoxemia and respiratory depression), recovery profiles (e.g., time to full alertness and satisfaction score), pain on injection, top-up dose requirement, type of surgery/procedure, dosage of study drugs, and country of publication, was systematically extracted. In cases of disagreement, a discussion was conducted to reach a consensus.

### 2.4 Outcomes and definitions

The dual primary outcomes were the success rate of sedation/anesthetic induction as well as the differences in sedation/induction time. Secondary outcomes included the risk of hemodynamic instability (e.g., hypotension), respiratory complications, recovery profiles (e.g., time to full alertness, risk of PONV, discharge time, satisfaction score), pain on injection, and top-up dose requirement. The definition and criteria used for each outcome was based on the definitions provided in the individual studies, rather than imposing a single standardized definition across all studies. For the current study, discharge time referred to the time from the end of procedures or/and the last instance of drug administration up until the fulfilment of discharge criteria according to the definition of individual studies.

### 2.5 Risk of bias and certainty of evidence

In accordance with the revised Cochrane risk of bias tool for randomized trials (RoB 2.0 tool) (Sterne et al., 2019), two reviewers conducted an independent assessment of the risk of bias for the included studies. Disagreements pertaining to the RoB assessment were resolved by a third reviewer. The quality of the included trials was categorized into three levels: “low risk of bias,” “some concerns,” and “high risk of bias,” based on six domains including the randomization process, deviations from the intended interventions, missing outcome data, measurement of the outcome, selective reporting of results, as well as the overall risk of bias.

The evaluation of the certainty of evidence was performed independently by the same two reviewers based on five criteria, namely, the risk of bias, inconsistency, indirectness, imprecision, and publication bias. In the event of disagreements, a third reviewer intervened to reach a final decision.

### 2.6 Strategy for data synthesis

The data was analyzed using the random-effects model to determine the pooled risk ratio (RR), mean difference (MD), or standardized mean difference (SMD). Additionally, the 95% confidence interval (CI) was reported for each outcome. To assess heterogeneity for each outcome, I^2^ statistics values of 50% or higher were deemed indicative of substantial heterogeneity as previously reported (Hung et al., 2022). Sensitivity analysis was employed to determine the reliability of primary and secondary outcomes through a leave-one-out approach. Potential publication bias was evaluated for outcomes reported in 10 or more studies/dataset using visual analysis of a funnel plot. Subgroup analysis was performed based on the clinical setting (i.e., sedation or anesthetic induction). Statistical analyses were performed using Review Manager (RevMan) or comprehensive Meta-Analysis (CMA) V3 software (Biostat, Englewood, NJ, United States). Statistical significance was set at a probability value (*p*) of less than 0.05.

## 3 Results

### 3.1 Study selection

**FIGURE 1:**
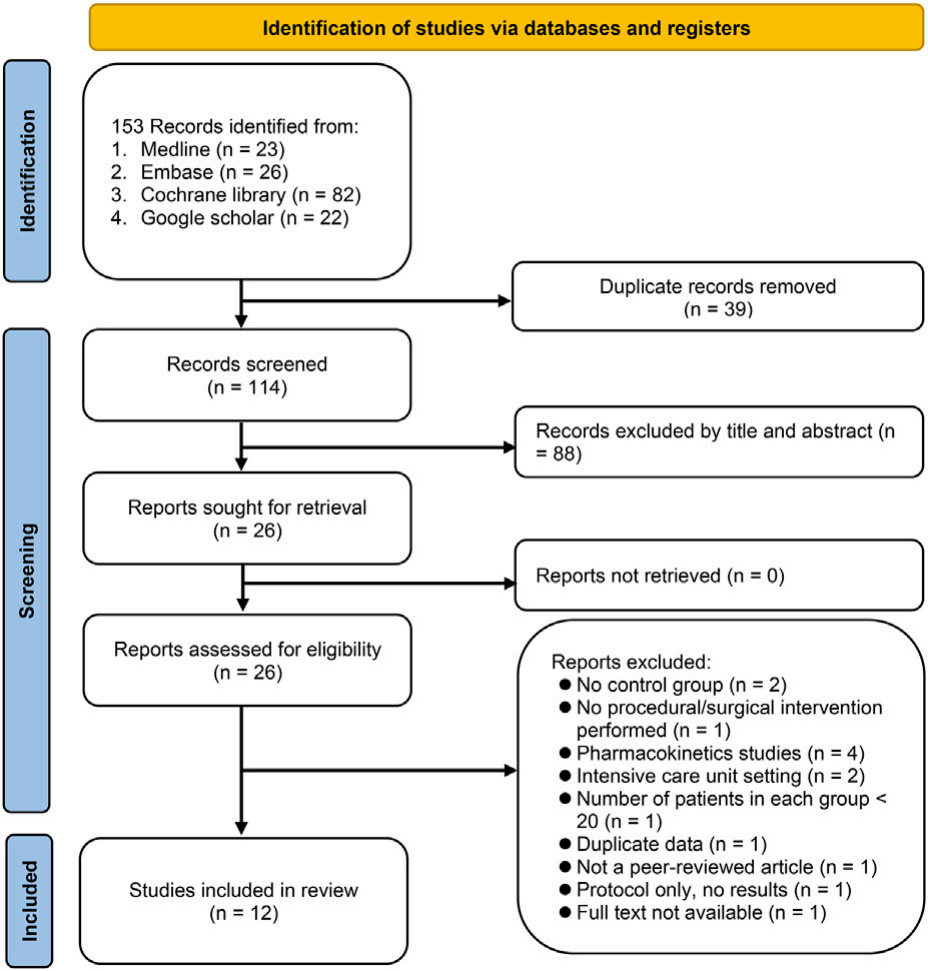
From a search across Medline, Embase, Cochrane library, and Google Scholar, 153 records were identified. After deduplication and preliminary screening, 88 of 114 articles were excluded. Of the 26 articles assessed in full, 14 were excluded for reasons including lack of control group, non-surgical patients, being pharmacokinetic studies, intensive care unit (ICU) setting, or having small sample sizes. Four more were excluded for duplicate data, being non-peer-reviewed, being only protocols, or unavailable full texts.

The literature search and selection process involving multiple databases, including Medline, Embase, Cochrane library, and the Google Scholar, resulted in the identification of a total of 153 records (Figure 1). After removing 39 duplicates, screening of the title and abstract of the remaining 114 records led to further exclusion of 88 articles. The full texts of the remaining 26 articles were assessed for eligibility, of which 14 were excluded due to: lack of a control group (2 studies), patients did not undergo any procedure or surgery (1 study), being pharmacokinetics studies (4 studies), conducted in an ICU setting (2 studies), and having fewer than 20 patients per group (1 study). An additional 4 studies were excluded for other reasons including duplicate data (1 study), non-peer-reviewed study (1 study), only a protocol (1 study), or full text not available (1 study). Finally, a total of 12 RCTs published between 2021 and 2023 were included in the current meta-analysis (Teng et al., 2021; Chen et al., 2022a; Chen et al., 2022b; Li et al., 2022; Luo et al., 2022; Qin et al., 2022; Wang et al., 2022; Wu et al., 2022; Liang et al., 2023; Man et al., 2023; Zhong et al., 2023; Zhu et al., 2023).

### 3.2 Characteristics and quality of studies

All twelve studies were conducted in China with the inclusion of a total of 1793 participants. Among the 12 RCTs, six employed ciprofol as a sedative for diverse procedures, including gastrointestinal (GI) endoscopy (*n* = 3) (Teng et al., 2021; Chen et al., 2022b; Li et al., 2022), fiberoptic bronchoscopy (*n* = 2) (Luo et al., 2022; Wu et al., 2022), and mixed procedures (GI endoscopy or fiberoptic bronchoscopy) (*n* = 1) (Zhong et al., 2023). The other six RCTs evaluated the efficacy of ciprofol as an induction agent in gynecological surgery (*n* = 2) (Chen et al., 2022a; Man et al., 2023), elective surgery (*n* = 3) (Wang et al., 2022; Liang et al., 2023; Zhu et al., 2023), and kidney transplantation (*n* = 1) (Qin et al., 2022). Of these six studies, three also use ciprofol as an agent for anesthetic maintenance (Qin et al., 2022; Liang et al., 2023; Man et al., 2023). The mean or median age of the participants ranged from 34 to 58 years. The male percentage varied from 0% to 54.3% with two studies focusing on female patients receiving gynecological surgery (Chen et al., 2022a; Man et al., 2023). The ASA physical status classification varied from I to IV with ASA II being the most common. Nine studies included patients with ASA I-II (Teng et al., 2021; Chen et al., 2022a; Chen et al., 2022b; Li et al., 2022; Wang et al., 2022; Wu et al., 2022; Liang et al., 2023; Man et al., 2023; Zhu et al., 2023), while two studies recruited patients with ASA I-III (Luo et al., 2022; Zhong et al., 2023). In contrast, one study only enrolled high-risk patients (i.e., ASA III-IV) undergoing kidney transplantation (Qin et al., 2022).

Among the 12 RCTs, three employed a three-arm study design to compare the efficacy of different dosages of ciprofol with propofol (Teng et al., 2021; Zhong et al., 2023; Zhu et al., 2023). The dosage of ciprofol and propofol employed in the studies varied widely. For sedation or anesthetic induction, the dosage of ciprofol ranged from 0.3 to 0.5 mg/kg in 10 studies (Teng et al., 2021; Chen et al., 2022a; Li et al., 2022; Luo et al., 2022; Qin et al., 2022; Wang et al., 2022; Wu et al., 2022; Liang et al., 2023; Man et al., 2023; Zhu et al., 2023), whereas one study implemented dosages of 6 and 8 mg/kg/h for sedation (Zhong et al., 2023). However, one study did not provide relevant information regarding ciprofol dosage (Chen et al., 2022b). On the other hand, the dosage of propofol ranged from 1.2 to 2.0 mg/kg for sedation or anesthetic induction in 11 studies (Teng et al., 2021; Chen et al., 2022a; Chen et al., 2022b; Li et al., 2022; Luo et al., 2022; Qin et al., 2022; Wang et al., 2022; Wu et al., 2022; Liang et al., 2023; Man et al., 2023; Zhu et al., 2023) with one study utilizing a dosage of 40 mg/kg/h for sedation (Zhong et al., 2023).

**FIGURE 2:**
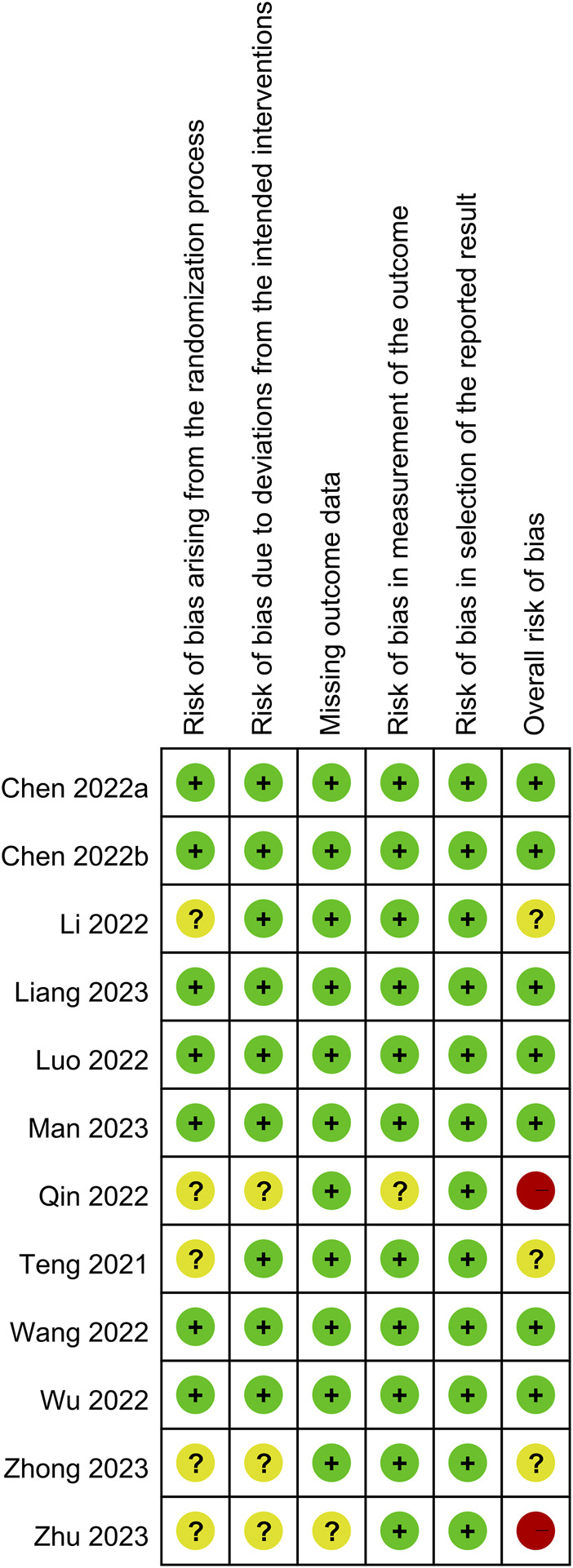
Risks of bias of the included randomized controlled trial. Seven randomized controlled trials (RCTs) were regarded as having a low overall risk of bias. In contrast, three RCTs raised concerns in some domains such as the risk of bias arising from the randomization process, which led to some concerns in the overall risk of bias. Notably, two RCTs were deemed to have a high overall risk of bias. Green indicates low risk of bias; yellow indicates some concerns or uncertain risk of bias; red indicates high risk of bias.

The results of an overall evaluation of all domains of bias for the 12 RCTs are summarized in Figure 2. Seven RCTs were regarded as having a low overall risk of bias (Chen et al., 2022a; Chen et al., 2022b; Luo et al., 2022; Wang et al., 2022; Wu et al., 2022; Liang et al., 2023; Man et al., 2023), indicating well-conducted studies with reliable results. In contrast, three RCTs (Teng et al., 2021; Li et al., 2022; Zhong et al., 2023) raised concerns in some domains such as the risk of bias arising from the randomization process, which led to some concerns in the overall risk of bias. Notably, two RCTs (Qin et al., 2022; Zhu et al., 2023) were deemed to have a high overall risk of bias, highlighting significant concerns regarding the validity and reliability of the findings.

### 3.3 Outcomes

#### 3.3.1 Primary outcomes: success rate and time required for successful anesthetic induction/sedation

**FIGURE 3:**
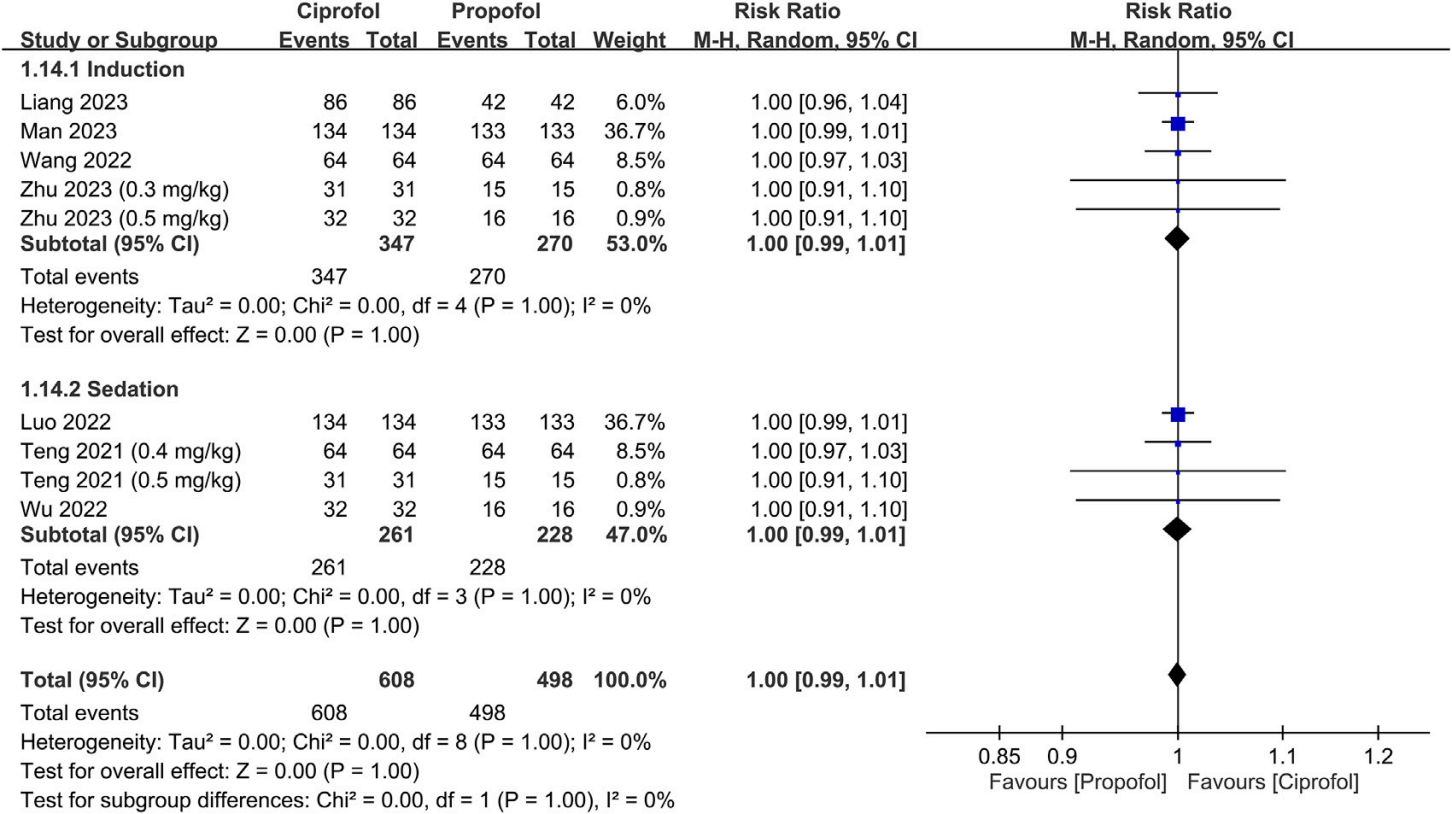
Forest plot comparing the success rate between the ciprofol and propofol groups. M-H, Mantel-Haenszel; CI, confidence interval.

**FIGURE 4:**
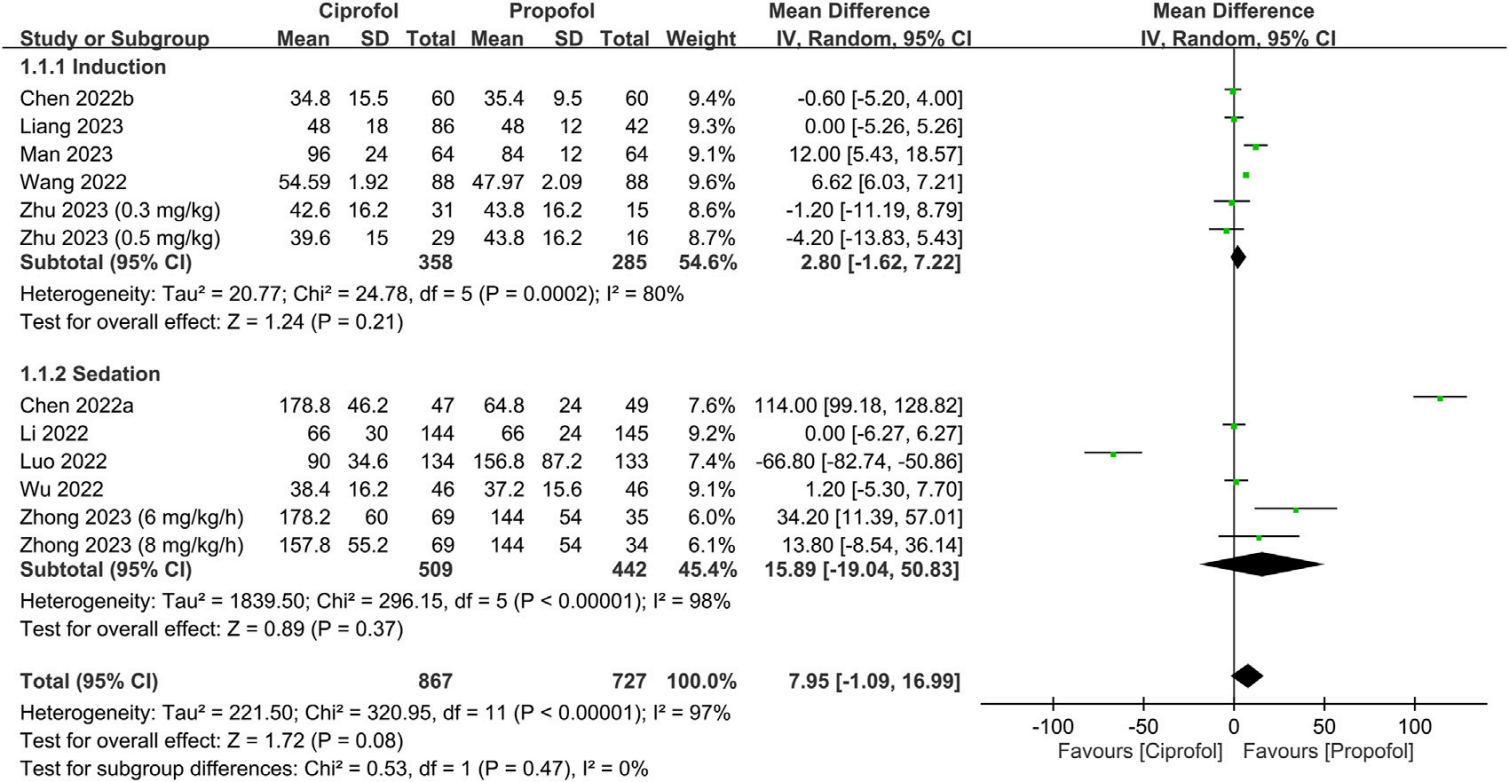
Forest plot showing the difference in time required for anesthetic induction/sedation between the ciprofol and propofol groups. IV, inverse variance; CI, confidence interval.

The success rate and time required for successful anesthetic induction/sedation are shown in Figures 3, 4, respectively. There were no differences in the success rate (RR = 1, 95% CI: 0.99 to 1.01, I^2^ = 0%, *p* = 1.0, 1,106 patients, sensitivity analysis: consistent) (Teng et al., 2021; Luo et al., 2022; Wang et al., 2022; Wu et al., 2022; Liang et al., 2023; Man et al., 2023; Zhu et al., 2023) and time required for successful anesthetic induction/sedation (MD = 7.95 s, 95% CI: −1.09 to 16.99, I^2^ = 97%, *p* = 0.08, 1,594 patients) (Chen et al., 2022a; Chen et al., 2022b; Li et al., 2022; Luo et al., 2022; Wang et al., 2022; Wu et al., 2022; Liang et al., 2023; Man et al., 2023; Zhong et al., 2023; Zhu et al., 2023) between the ciprofol and propofol groups. Sensitivity analysis revealed a shorter time to achieve anesthetic induction/sedation with the use of propofol than that with ciprofol when one study was removed (Luo et al., 2022). Similarly, subgroup analysis based on the clinical setting (i.e., sedation or anesthetic induction) demonstrated no difference in success rate or time for successful anesthetic induction/sedation between the two groups. The levels of certainty on the success rate and time required for successful anesthetic induction/sedation were evaluated as high and moderate, respectively.

#### 3.3.2 Secondary outcomes: top-up doses required, risk of adverse event, and recovery parameters

**FIGURE 5:**
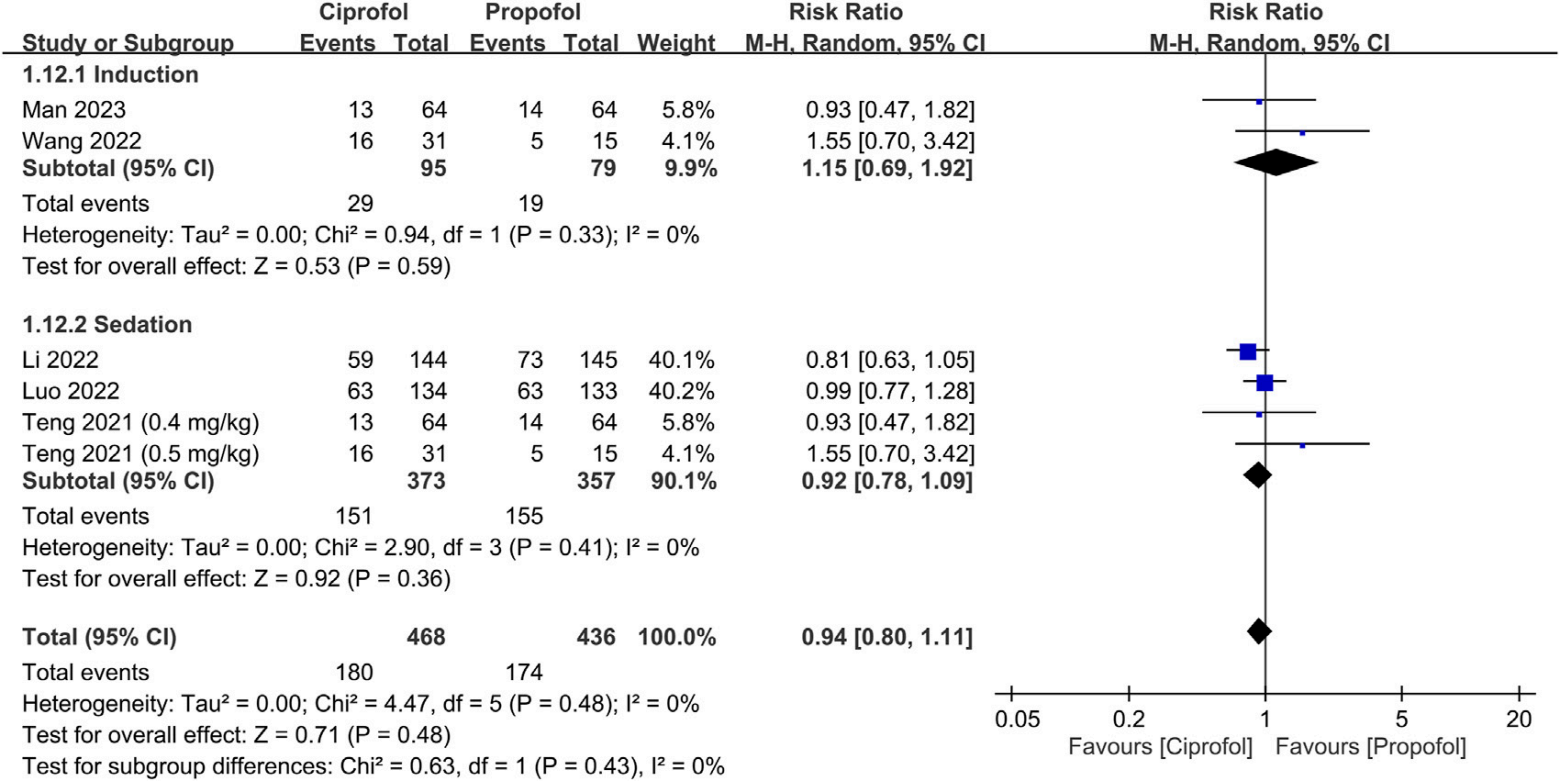
Forest plot comparing the risk of top-up dose requirement between the ciprofol and propofol groups. M-H, Mantel-Haenszel; CI, confidence interval.

**FIGURE 6:**
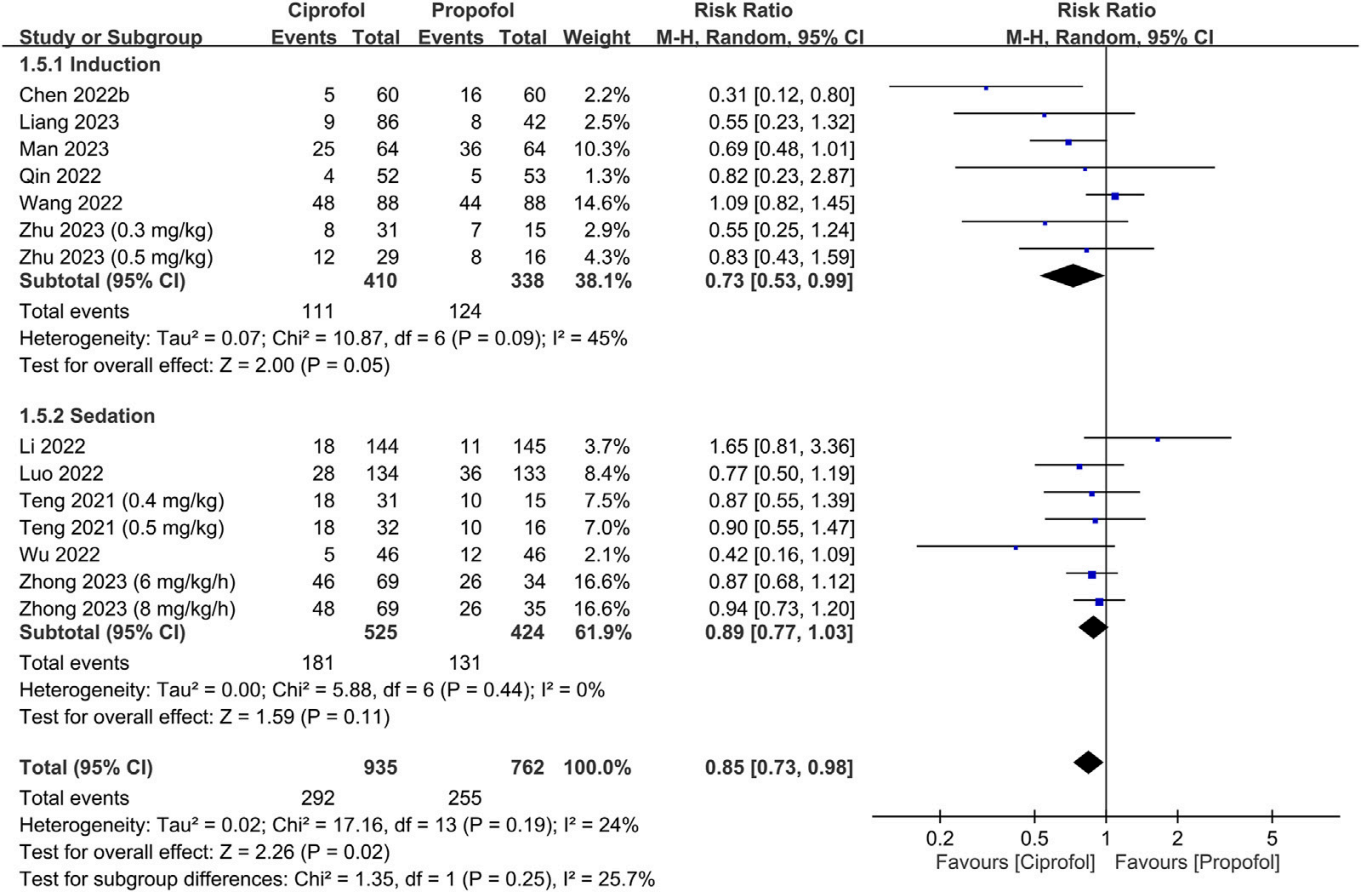
Forest plot comparing the risk of hypotension between the ciprofol and propofol groups. M-H, Mantel-Haenszel; CI, confidence interval.

**FIGURE 7:**
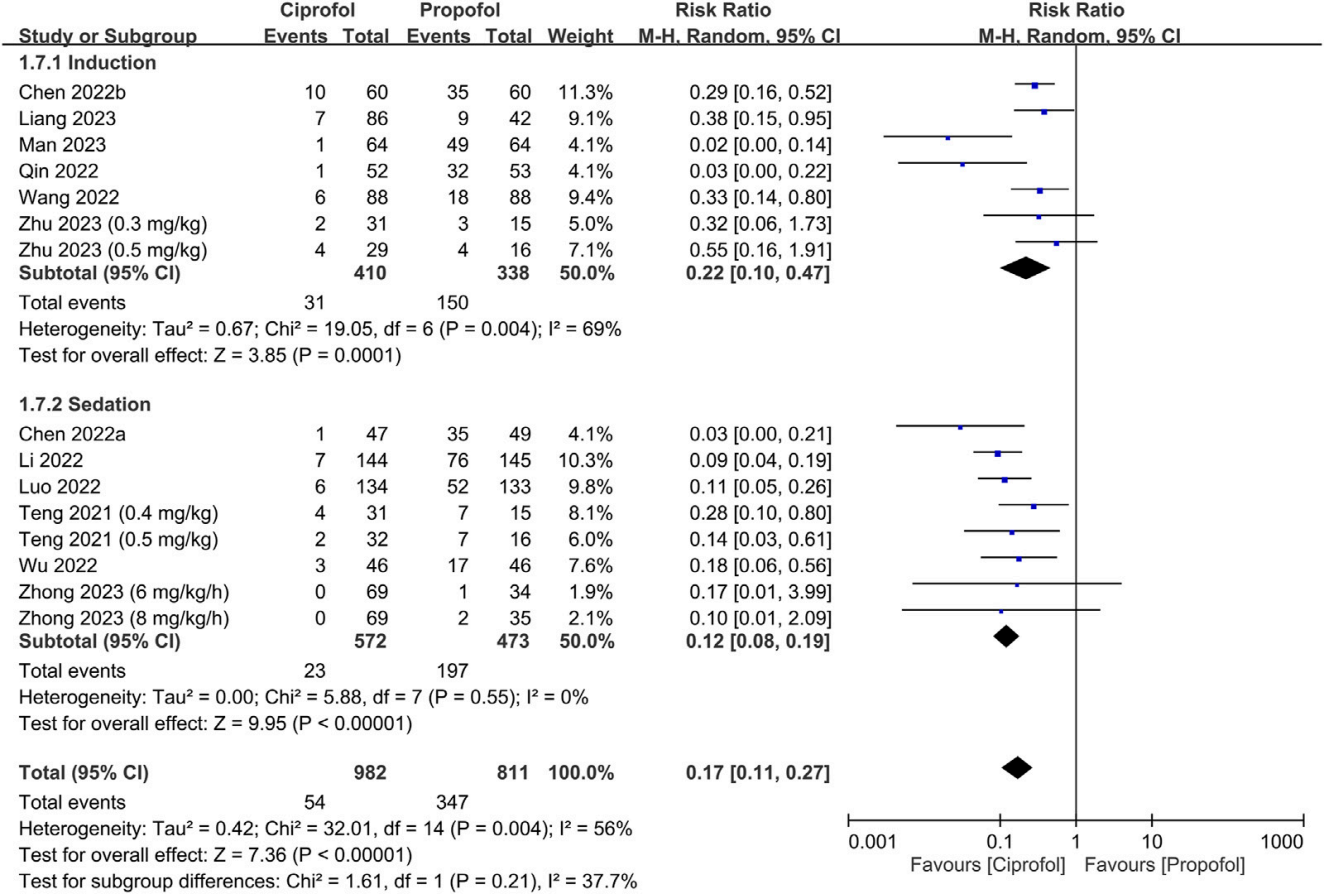
Forest plot comparing the risk of pain on injection between the ciprofol and propofol groups. M-H, Mantel-Haenszel; CI, confidence interval.

The risks of top-up dose requirement, cardiopulmonary complications, and pain on injection, as well as recovery parameters between the ciprofol and propofol groups are summarized in Table 2. There were no differences in the risks of top-up dose requirement (RR = 0.94, 95% CI: 0.80 to 1.11, *p* = 0.48) (Figure 5), bradycardia (RR = 0.94, 95% CI: 0.73 to 1.23, *p* = 0.67) (Supplementary Figure S1), tachycardia (RR = 0.83, 95% CI: 0.34 to 2.04, *p* = 0.68) (Supplementary Figure S2), hypertension (RR = 1.28, 95% CI: 0.88 to 1.86, *p* = 0.2) (Supplementary Figure S3), pulmonary complications (RR = 0.78, 95% CI: 0.51 to 1.19, *p* = 0.24) (Supplementary Figure S4), and PONV (RR = 0.85, 95% CI: 0.35 to 2.06, *p* = 0.72) (Supplementary Figure S5), as well as discharge time (MD = 1.39 min, 95% CI: −0.45 to 3.22, *p* = 0.14) (Supplementary Figure S6) and satisfaction score (SMD = 0.23, 95% CI:−0.10 to 0.56, *p* = 0.16) (Supplementary Figure S7) between the two groups. In contrast, the risks of hypotension (RR = 0.85, 95% CI: 0.73 to 0.98, *p* = 0.02) (Figure 6) and pain on injection (RR = 0.17, 95% CI: 0.11 to 0.27, *p* < 0.00001) (Figure 7) were lower in the ciprofol group compared to those in the propofol group. The time to full alertness was statistically shorter by a clinically non-significant 0.66 min (95% CI: 0.14 to 1.18, *p* = 0.01) (Supplementary Figure S8) in patients being given propofol than those receiving ciprofol.

The results of sensitivity analysis showed a wide variation with regard to the risk of hypotension, time to full alertness, discharge time, and satisfaction score, suggesting a lack of consistency. Conversely, sensitivity analysis of other outcomes demonstrated consistent findings.

Table 1 presents a summary of the certainty of evidence pertaining to different outcomes of the current study. Notably, a high level of certainty was assigned to the outcomes on the risks of top-up dose requirement, bradycardia, hypertension, and pain on injection. Conversely, the certainty of evidence for outcomes such as tachycardia, hypotension, hypoxemia, time to full alertness, and PONV was considered moderate. In contrast, the certainty of evidence was deemed low for discharge time and satisfaction score.

**TABLE 1:** Study characteristics.

	Clinical setting	Mean or median age (years)	N	Male (%)	ASA I/II/III/IV	Procedures	Ciprofol (mg/kg)	Propofol (mg/kg)	Country
Chen et al., 2022a	Sedation	41 vs. 43	96	40.6	86/10/0/0	GI scopy	NA	1.5∼2	China
Chen et al., 2022b	AI	34 vs. 34	120	0	66/54/0/0	GYN surgery	0.4	2	China
Li et al., 2022	Sedation	44 vs. 44	289	40.8	233/56/0/0	GI scopy	0.4	1.5	China
Liang et al., 2023	AI	39 vs. 41	128	25.8	70/58/0/0	Elective surgery	0.4	2	China
							0.8 mg/kg/h	5 mg/kg/h	
Luo et al., 2022	Sedation	47 vs. 47	267	50.6	113/150/4/0	FB	0.4	2	China
Man et al., 2023	AI	42 vs. 44	128	0	32/96/0/0	GYN surgery	0.5	2	China
Qin et al., 2022	AI	39 vs. 41	105	34.3	0/0/86/19	KT	0.4	2.0	China
Teng et al., 2021	Sedation	46 vs. 48 vs. 48	94	44.7	75/19/0/0	GI scopy	0.4; 0.5	2.0	China
Wang et al., 2022	AI	39 vs. 41	176	35.8	99/77/0/0	Elective surgery	0.4	2.0	China
Wu et al., 2022	Sedation	58 vs. 57	92	54.3	18/74/0/0	FB	0.3	1.2	China
Zhong et al., 2023	Sedation	57 vs. 58 vs. 57	207	53.6	38/150/19/0	GI scopy/FB	6 mg/kg/h; 8 mg/kg/h	40 mg/kg/h	China
Zhu et al., 2023	AI	45 vs. 47 vs. 45	91	50.5	38/53/0/0	Elective surgery	0.3; 0.5	2	China

AI, anesthetic induction; FB, fiberoptic bronchoscopy; KT, kidney transplantation; GYN, surgery, Gynecological surgery; GI, gastrointestinal; ASA, American society of anesthesiologists; NA, not available.

## 4 Discussion

This meta-analysis, which included 12 RCTs published from 2021 to 2023, found no significant differences in success rate and time for anesthetic induction/sedation between the ciprofol and propofol groups. Similarly, the risks of top-up dose requirement, cardiopulmonary complications (i.e., bradycardia, tachycardia, hypertension, hypoxemia/pulmonary depression), and PONV, as well as discharge time and satisfaction score did not differ significantly between the two groups. However, the ciprofol group had lower risks of hypotension and pain on injection than the propofol group. Although the time to full alertness was statistically shorter in the propofol group, it was clinically insignificant. This meta-analysis is the first to investigate the efficacy and safety of ciprofol use as an induction/sedative agent in clinical practice.

Sedative medications such as propofol are commonly used in a variety of medical settings, including diagnostic, surgical, and other therapeutic procedures, as well as anesthetic induction and critical care (Ghojazadeh et al., 2019; Freitas et al., 2022; Guo et al., 2023). Despite the ability of sedatives to provide relief from procedure-related anxiety and discomfort through the induction of sedation and anxiolysis, safety concerns remain an important issue as they can cause varying degrees of depression in the respiratory and cardiovascular systems as well as a loss of airway reflexes (Godoroja-Diarto et al., 2022; Sneyd et al., 2022; Hara et al., 2023). The efficacy and safety of sedatives can be evaluated based on their possession of optimal properties for clinical use, including a rapid onset of action, minimal cardiopulmonary depression, rapid and smooth awakening from sedation, minimal cognitive dysfunction, and a low risk of PONV (Khorsand et al., 2022; Thomson et al., 2010; Minami and Takigawa, 2023). In addition to a low risk of adverse events, sedatives should be easy to administer and monitor with predictable effects to minimize possible medication errors and risk of complications, thereby improving patient outcomes (Gan, 2006; Manzi et al., 2021).

Similar to propofol, ciprofol functions as a positive allosteric modulator of GABA-A receptors, binding to them and augmenting the inhibitory effect of the neurotransmitter GABA, thereby inducing sedative, hypnotic, and anesthetic effects (Lu et al., 2023). Ciprofol is estimated to possess approximately 4–5 times greater potency than propofol, although its precise potency ratio is still being fully characterized (Qin et al., 2017). After intravenous administration of ciprofol, its anesthetic effects begin within 30–60 s (Lu et al., 2023). Owing to its high lipophilicity, ciprofol undergoes a rapid distribution phase characterized by extensive tissue distribution, including easy passage across the blood-brain barrier, thereby facilitating its central nervous system effects (Lu et al., 2023). Ciprofol undergoes substantial hepatic metabolism, primarily through glucuronidation, resulting in a terminal half-life of approximately 2 h (Lu et al., 2023).

Several studies examining patients undergoing gastrointestinal endoscopy or fiberoptic bronchoscopy have indicated comparable efficacy as a sedative agent between ciprofol at a dose of 0.4 mg/kg and propofol at 2.0 mg/kg (Teng et al., 2021; Luo et al., 2022). In the present meta-analysis, the regimens of ciprofol at 0.4 mg/kg and propofol at 2.0 mg/kg predominated across the included studies. The pooled results showed no significant differences in success rate between the two groups, suggesting comparable probability of achieving successful anesthetic induction/sedation between the two agents. Furthermore, our subgroup and sensitivity analyses demonstrated consistent findings. Moreover, there were no significant differences in the time required for successful anesthetic induction/sedation and the risk of top-up dose requirement between the two groups, further supporting similar efficacy of both agents for anesthetic induction and sedation. The high degree of heterogeneity (I^2^ = 97%) on the time required for successful anesthetic induction/sedation may be partially attributed to the variations in sedative dosage and procedure (e.g., fiberoptic bronchoscopy or gastrointestinal endoscopy) across our included studies.

Minimal cardiopulmonary depression is a prerequisite for drugs utilized in sedation and anesthetic induction. Despite no significant difference in the risks of bradycardia, tachycardia, hypertension, and hypoxemia between the two groups (Table 2), individuals who received ciprofol exhibited a lower risk of hypotension compared to those being given propofol. Prior studies have indicated that hypotension is linked to a heightened risk of myocardial and renal injury (Ahuja et al., 2020), as well as elevated mortality rates (Wesselink et al., 2018). Furthermore, the longer the exposure and the lower the blood pressure, the greater the risk of mortality (Wesselink et al., 2018). Therefore, our finding of a lower risk of hypotension associated with ciprofol may suggest a safety profile superior to that of propofol. It is noteworthy that the enrollment of relatively healthy patients (e.g., ASA I-II) in the majority of studies included in the current meta-analysis may in part explain the subtle disparity between ciprofol and propofol, which may be more conspicuous in the diseased population. Indeed, only one study recruited patients with ASA III to IV; hence, the benefits of ciprofol in the management of patients with severe comorbidities warrant further evaluations.

**TABLE 2:** Effect Estimate, subgroup analysis, sensitivity, risk of publication bias, and certainty of evidence of secondary outcomes.

Overall outcome or subgroup	Studies	Participants	Effect estimate (MD, SMD, RR)	*p*-value	I^2^ ^a^	Sensitivity analysis	PB	Favor^b^	Certainty of evidence
Top-up dose requirement	6	904	RR 0.94 (0.80, 1.11)	0.48	0%	Consistent	-	NS	High
Induction	2	174	RR 1.15 (0.69, 1.92)	0.59	0%	-	-	NS	-
Sedation	4	730	RR 0.92 (0.78, 1.09)	0.36	0%	-	-	NS	-
Risk of cardiopulmonary complications and pain on injection									
Bradycardia	14	1,697	RR 0.94 (0.73, 1.23)	0.67	0%	Consistent	Low	NS	High
Induction	7	748	RR 0.82 (0.58, 1.15)	0.25	0%	-	-	NS	-
Sedation	7	949	RR 1.15 (0.77, 1.72)	0.5	0%	-	-	NS	-
Tachycardia	5	515	RR 0.83 (0.34, 2.04)	0.68	0%	Consistent	-	NS	Moderate
Induction	5	515	RR 0.83 (0.34, 2.04)	0.68	0%	-	-	NS	-
Sedation	0	0	RR Not estimable	-	-	-	-	-	-
Hypertension	8	814	RR 1.28 (0.88, 1.86)	0.2	0%	Consistent	-	NS	High
Induction	5	515	RR 1.30 (0.86, 1.97)	0.22	0%	-	-	NS	-
Sedation	3	299	RR 1.20 (0.52, 2.82)	0.67	0%	-	-	NS	-
Hypotension	14	1,697	RR 0.85 (0.73, 0.98)	0.02	24%	Inconsistent	Low	C	Moderate
Induction	7	748	RR 0.73 (0.53, 0.99)	0.05	45%	-	-	C	-
Sedation	7	949	RR 0.89 (0.77, 1.03)	0.11	0%	-	-	NS	-
Respiratory complications^c^	7	1,330	RR 0.78 (0.51, 1.19)	0.24	0%	Consistent	-	NS	High
Hypoxemia	5	855	RR 0.81 (0.47, 1.40)	0.45	13%	-		NS	Moderate
Pulmonary depression	4	475	RR 0.7 (0.31, 1.59)	0.39	0%	-		NS	
Pain on injection	15	1793	RR 0.17 (0.11, 0.27)	<0.00001	56%	Consistent	low	C	High
Induction	7	748	RR 0.22 (0.10, 0.47)	0.003	69%	-	-	C	-
Sedation	8	1,045	RR 0.12 (0.08, 0.19)	0.0009	0%	-	-	C	-
Characteristics of recovery									
Time to full alertness	11	1,296	MD 0.66 (0.14, 1.18)	0.01	34%	Inconsistent	low	P	Moderate
Induction	4	347	MD 0.67 (−0.04, 1.37)	0.06	0%	-	-	NS	-
Sedation	7	949	MD 0.70 (−0.08, 1.48)	0.08	58%	-	-	NS	-
PONV	5	639	RR 0.85 (0.35, 2.06)	0.72	0%	Consistent	-	NS	Moderate
Induction	4	372	RR 0.92 (0.37, 2.31)	0.86	0%	-	-	NS	-
Sedation	1	267	RR 0.33 (0.01, 8.05)	0.5	-	-	-	-	-
Discharge time	7	792	MD 1.39 (−0.45, 3.22)	0.14	80%	Inconsistent	-	NS	Low
Induction	1	128	MD 0.30 (−1.65, 2.25)	0.76	-	-	-	NS	-
Sedation	6	664	MD 1.57 (−0.48, 3.62)	0.13	81%	-	-	NS	-
Satisfaction scores	7	814	SMD 0.23 (−0.10, 0.56)	0.16	74%	Inconsistent	-	NS	Low
Patients	4	453	SMD 0.49 (0.28, 0.70)	<0.00001	0%	-	-	C	-
Anesthesiologists	3	361	SMD −0.04 (−0.29, 0.20)	0.73	10%	-	-	NS	-

^a^ Heterogeneity; PB, publication bias; NS, no significance between both groups; MD, mean difference; SMD, standardized mean difference; RR, risk ratio; PONV, postoperative nausea and vomiting.

^b^ Favor Ciprofol (C) or propofol (P).

^c^ Data from patients receiving sedation.

In addition to the risk of dose-dependent hemodynamic instability, propofol administration is frequently associated with injection pain, which is a commonly reported adverse reaction. Such discomfort, which is likely due to the high concentration of propofol, can result in anxiety and body movements during drug administration (Tan and Onsiong, 1998; Marik, 2004). Compared with propofol, the demonstration of a lower risk of injection pain linked to ciprofol may be another benefit of its clinical use. With regard to recovery characteristics, no significant differences in the risk of PONV, discharge time, and satisfaction score were observed between the two agents. Although the time to full alertness was slightly shorter with propofol than ciprofol (MD: 0.66 min), the difference was not considered clinically relevant. Surprisingly, despite limited data from only four studies, the apparently higher degree of satisfaction associated with ciprofol than propofol may be partially attributed to the lower incidence of injection pain linked to ciprofol use. Focusing on the effects of sedatives on cognitive function, despite the reported impacts of different sedatives on subsequent psychomotor and cognitive function as well as explicit and implicit memory (Sarasin et al., 1996; Wang et al., 2019), the effects of both agents on those recovery characteristics were not evaluated due to a lack of related information in all studies. Therefore, further investigations are needed to address these issues to provide clinical implications for better outpatient care.

Several key factors should be considered when comparing propofol and ciprofol. First, propofol and ciprofol have different potencies, with ciprofol estimated to be 4–5 times more potent than propofol (Qin et al., 2017). This should be considered when calculating doses for comparison. Equating the dosages on a simple mg/kg basis would fail to provide an accurate assessment. Second, while the two drugs have similar pharmacokinetic properties, such as rapid onset and short duration of action, there are some differences in parameters, such as clearance and volume of distribution, that can impact the comparison. Third, patient factors, including age, health status, and type of procedure, can influence drug performance and side-effect profiles, potentially leading to variations in comparisons across different patient populations.

Through a comprehensive synthesis of existing evidence, our meta-analysis established that ciprofol presented comparable efficacy to propofol for sedation and anesthetic induction, supported by equivalent success rates and induction times. Notably, ciprofol offers safety benefits, including reduced hypotension risk and lower injection pain incidence compared to propofol. In addition, the recovery characteristics and overall safety profiles were generally similar. Accordingly, ciprofol can be considered when propofol is contraindicated or not well tolerated. The reduced risk of hypotension suggests that ciprofol may be preferred in patients at high risk of complications from hypotension, such as the elderly or those with cardiovascular disease. For patients experiencing pain upon propofol injection, switching to ciprofol is an option to improve comfort. Dosage adjustments between the two drugs must account for the higher potency of ciprofol than that of propofol. Equivalent doses may lead to over-sedation with ciprofol. Monitoring for respiratory and cardiovascular side effects is still required as with other sedative medications.

The current meta-analysis was associated with several limitations that may impact the extrapolation and reliability of its findings. First, the fact that all of the included studies were conducted in China may limit extrapolation of the results to other populations. Second, the relatively narrow age range of the study participants (i.e., 34–58 years) may restrict the applicability of the findings to other age groups. Third, the wide variation in ciprofol and propofol dosages across the included studies may introduce heterogeneity that biased our results. Fourth, the diversity in anesthetic techniques for both sedation and anesthetic induction in the included trials may be another source of heterogeneity that could impede comparability of the findings between studies. Fifth, the recruitment of patients with different ASA physical status across the studies may obscure the significance of our findings. For instance, patients belonging to higher ASA classes, who were recruited in one of our included trials, may have more comorbidities that could affect their response to sedation or anesthesia compared to relatively healthy individuals. Finally, the small sample sizes in some studies may limit the power for discerning significant differences or associations between the variables of interest.

In conclusion, this meta-analysis of 12 RCTs including a total of 1,793 participants showed no significant differences in success rate and time for anesthetic induction/sedation between the ciprofol and propofol groups. The risks of various adverse events also did not significantly differ between the two groups, except for lower risks of hypotension and pain on injection in the ciprofol group. Despite the statistically shorter time to full alertness associated with propofol use, it was not of clinical significance. Future studies are warranted to evaluate the efficacy and safety of ciprofol in specific patient populations, such as pediatric or elderly patients.

## Data Availability

The original contributions presented in the study are included in the article/Supplementary Material, further inquiries can be directed to the corresponding author.
