# Supplementary material for: A systematic review and meta-analysis comparing the efficacy and safety of ciprofol (HSK3486) versus propofol for anesthetic induction and non-ICU sedation
Source: Front Pharmacol. 2023 Sep 25;14:1225288. doi: 10.3389/fphar.2023.1225288 (PMC10561285; doi:10.3389/fphar.2023.1225288)
Supplement: Supplementary file 1 [file Table1.docx]

**Supplemental Table 1.** Search strategies for Medline (OVID)

| 1 | ("Sedation" or "Sedative" or "Deep sedation" or "procedural sedation" or "depression of consciousness" or "sedative" or "Conscious sedation" or "Moderate sedation" or "General anesthesia*" or "Anesthesia*" or "tracheal intubation" or "laryngeal mask airway" or "anesthetic induction").mp. |
| --- | --- |
| 2 | exp "Deep Sedation"/ or exp "Anesthesia, General"/ or exp "Conscious Sedation"/ |
| 3 | ("HSK3486" or "ciprofol").mp. |
| 4 | (1 or 2) and 3 |
| 5 | 4 and (((randomized controlled trial or controlled clinical trial).pt. or randomi*ed.ab. or placebo.ab. or drug therapy.fs. or randomly.ab. or trial.ab. or groups.ab.) not (exp animals/ not humans.sh.)) |

**Supplemental Figure 1.** Forest plot comparing the risk of bradycardia between the ciprofol and propofol groups. M-H: Mantel-Haenszel, CI: confidence interval.

**
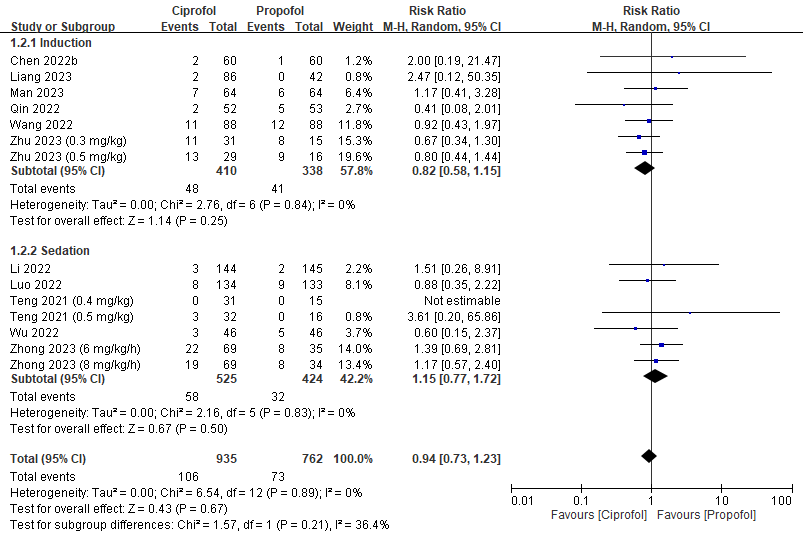
**

**Supplemental Figure 2.** Forest plot comparing the risk of tachycardia between the ciprofol and propofol groups. M-H: Mantel-Haenszel, CI: confidence interval.

**
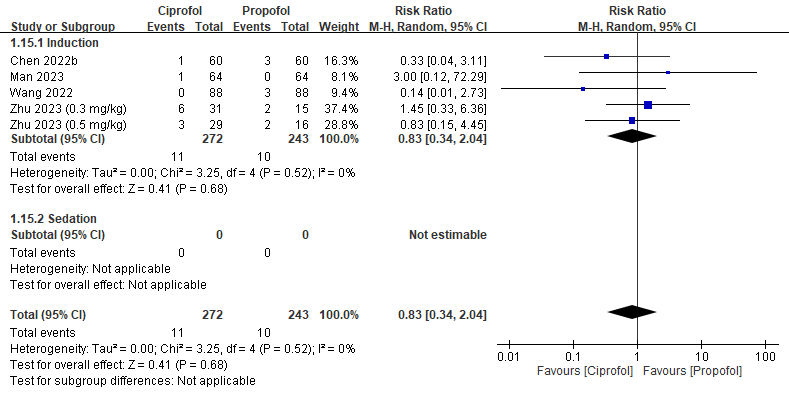
**

**Supplemental Figure 3.** Forest plot comparing the risk of hypertension between the ciprofol and propofol groups. M-H: Mantel-Haenszel, CI: confidence interval.

**
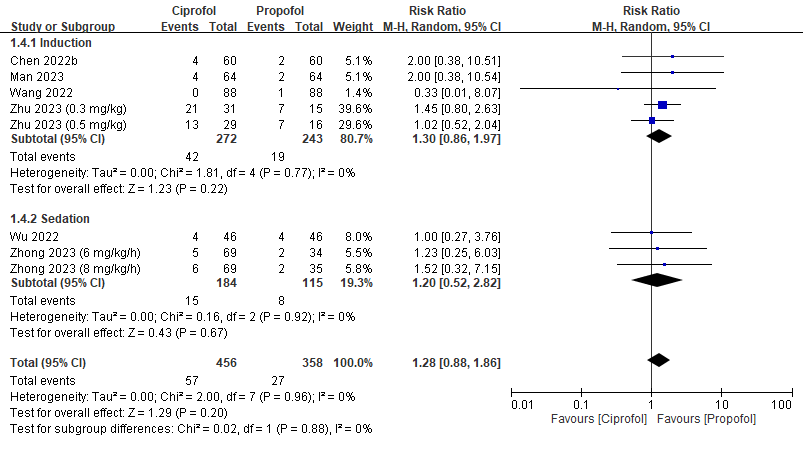
**

**Supplemental Figure 4.** Forest plot comparing the risk of respiratory complications between the ciprofol and propofol groups. M-H: Mantel-Haenszel, CI: confidence interval.

**
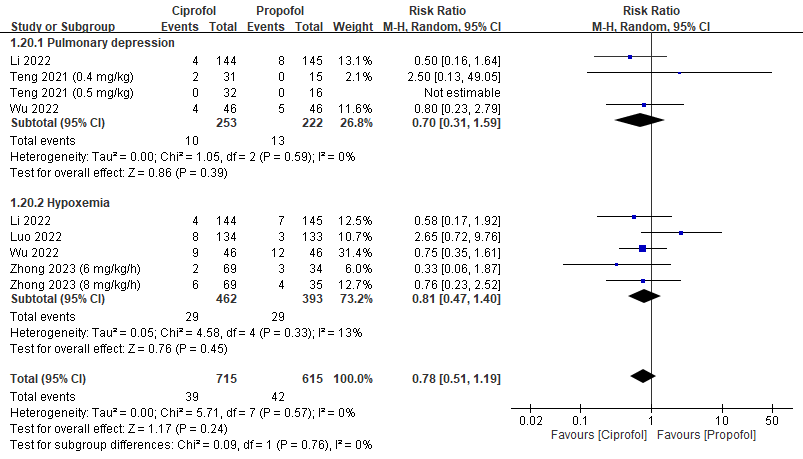
**

**Supplemental Figure 5.** Forest plot comparing the risk of postoperative nausea and vomiting between the ciprofol and propofol groups. M-H: Mantel-Haenszel, CI: confidence interval.


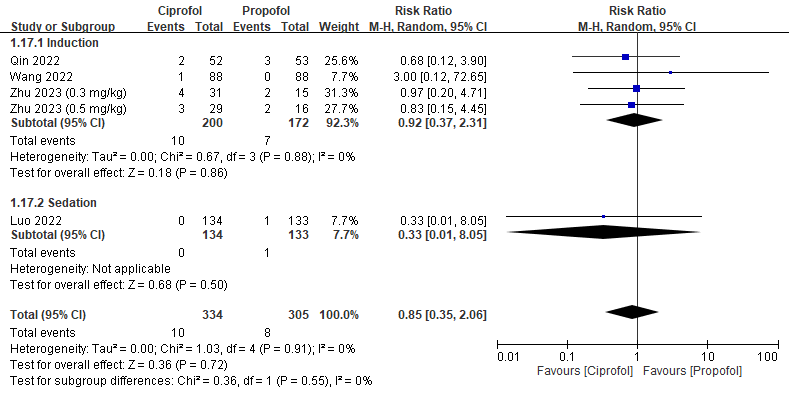


**Supplemental Figure 6.** Forest plot comparing the discharge time between the ciprofol and propofol groups. IV: inverse variance; CI: confidence interval.


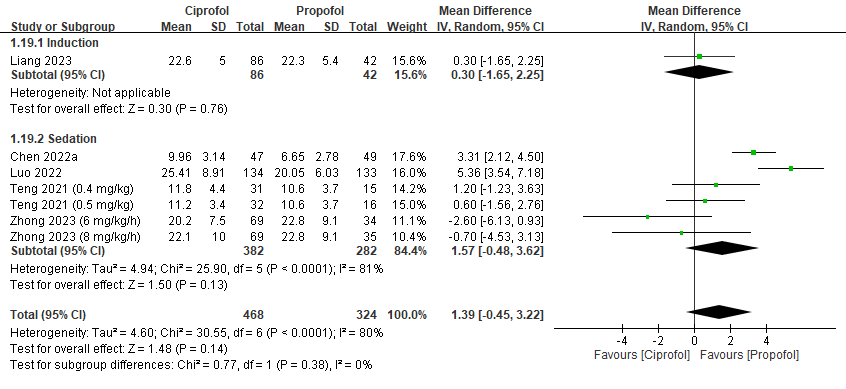


**Supplemental Figure 7.** Forest plot comparing the satisfaction scores between the ciprofol and propofol groups. IV: inverse variance; CI: confidence interval.


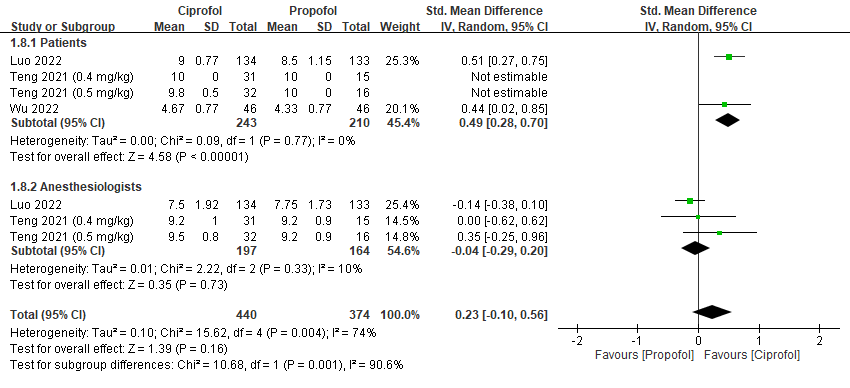


**Supplemental Figure 8.** Forest plot comparing the time to full alertness between the ciprofol and propofol groups. IV: inverse variance; CI: confidence interval.

**
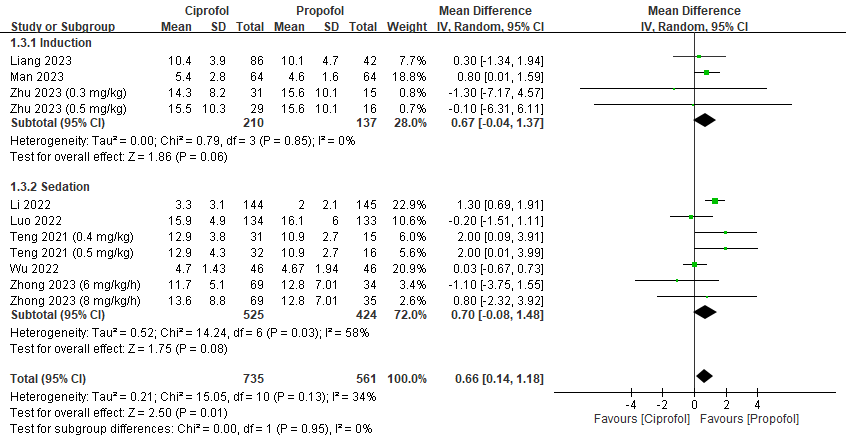
**
